# Peer effects on compliance with extortive requests

**DOI:** 10.1371/journal.pone.0231879

**Published:** 2020-04-24

**Authors:** Giulia Andrighetto, Daniela Grieco

**Affiliations:** 1 Institute of Cognitive Science and Technology, Italian National Research Council, Rome, Italy; 2 Malardalen University, Vasteras, Sweden; 3 Institute for Future Studies, Stockholm, Sweden; 4 Department of Law, University of Milan, Milan, Italy; Universidad Loyola Andalucia Cordoba, SPAIN

## Abstract

We conduct laboratory experiments to study peer effects on compliance with extortive requests. To this aim, we use an “extortion game” with multiple victims. In agreement with our hypothesis, our results show that when the information on peers’ behavior is available, compliance with appropriative requests is triggered by conformism among victims rather than by punishment. Moreover, we find that extorted sums are rather small, requests are proportional to the victim’s earnings, similar across victims, and are significantly lower when the extorter self-selects into this role. Punishment is rare, but effective. Finally, our results indicate that fairness concerns matter even in a context of extra-legal taxation, shaping both extorters’ requests and victims’ compliance.

## Introduction

Human interactions are often characterized by antisocial behavior. Among the possible forms it assumes, extortion is largely widespread, either as extra-legal taxation in criminal contexts or in the subtler shape of non-monetary appropriative requests within groups or organizations. Extortion is the continuous, regular and systematic demand for money or favors by a criminal or (more usually) a criminal organization [1].

The payment of extortion money constitutes one of the most important activities of criminal organizations: the revenues from extortion represent a source of economic income, typically used to sustain the families of convicted affiliates [2–3]. Obedience to these appropriative requests has been mainly explained as the result of threats, coercion and violence: “the extortive activity is effective because its victims know in advance that there is the possibility to suffer violent retaliations when the request is not accepted” [4–5]. Coercion is widely diffused also in the workplace, where subordinates can be victim of exploitation of effort and value, or excluded from opportunities [6].

In this work, we propose that apart from the principal's punishment, peers’ behavior has an effect on whether or not an individual complies with the appropriative request. Our results support this conjecture. The behavior of others has been shown to play an important role in affecting behavior in a wide variety of contexts, such as littering, taxation, voting and cooperation in general [7–10]. Individuals are more likely to engage in a behavior if they believe that others will engage in it as well. One explanation for this effect is that people tend to imitate peers’ behavior because they believe that is an expression of the social norms of their group, namely what people believe to be normal in the group, that is, believed to be a typical action, an appropriate action, or both [11–16].

Social norms have been shown to affect many aspects of our lives, from the act that we leave tips in restaurants to how we greet each other. Interestingly they can make stable any type of behavior: those that are socially desirable, like cooperation [17] and reciprocity [18], but also highly undesirable for society, like foot binding [19] and corruption [20–21]. There is anecdotal evidence that complying with extortive requests is the expected behaviour for people leaving in certain areas, where refusing to pay extortion would be punished not only by the criminal organization but also by peers through social ostracism and reprobation.

In this work, we examine experimentally the hypothesis that the expectation on what others do–what is typically referred to as a descriptive norm—affects people’s willingness to comply with extortive requests.

To test it, we experimentally analyze variants of the extortion game of [22]. Our experiment has been designed specifically to investigate repeated interaction among subjects where one of them can appropriate part of the other two subjects’ earnings. The design presents a series of features that have never been investigated in the same game all together. We use a “take” frame instead of the typical “give frame” used in ultimatum, dictator and public goods games usually studied to explore fairness, equity and reciprocity. Furthermore, we allow for the presence of multiple potential victims of appropriation, information sharing between them, and repeated interaction, to be able to test our prediction that the victim's behavior has an effect on whether or not her peer complies with extortive request. Finally, the design makes possible to manipulate the symmetry in the distribution of resources, and subjects’ possibility to self-select into the role of extorter or victim. Both aspects deal with the dimension of the personal responsibility of behaving antisocially in general, and making appropriative requests in this specific context. The former feature of the design allows us to contrast economic and non-economic motivations for appropriative behavior: contextual factors such as uneven opportunities of raising money can be used by extorters as a self-justification for mean behavior and then result in higher requests. The latter is meant to examine whether extortive dynamics can emerge endogenously without forcing them with assigned roles.

To anticipate our results, in agreement with our hypothesis, we show that when the information on peers’ behavior is available, compliance is triggered by conformism among victims rather than by punishment. Moreover, we find that the extorted sums are rather small, the extorter’s requests are proportional to the victim’s earnings, similar across victims, and are significantly lower when the extorter self-selects into this role and when he has his own source of earnings. Punishment is rare, but plays an important role in increasing victims’ compliance to the appropriative request. Finally, the perceived fairness of the extortive request is significantly and positively correlated with the request made by the extorter and shapes victims’ compliance with the request (the less the request is perceived as fair by the victim, the lower the level of compliance).

### Previous research

[22] represents the first work introducing the “extortion game” to explore the reasons why a principal may punish non-compliance of his potential victim and its effect on people willingness to comply with his appropriate request. In [22], pairs of subjects with asymmetric lump-sum endowment interact for a finite number of periods: in each round, one subject of type “P” in the pair demands cash transfer from her peer of type “A”, who can comply or refuse; after refusals P may punish A. Although the theoretical prediction is subjects of type “P” never punishing, and subjects of type “A” refusing any positive demand, the results show that P punishes increasingly often and severely as she gains experience; most As comply with P’s demands.

[23] studies the relationship between hierarchy and coercion, and presents a game with repeated interactions between a senior worker and a junior one. The two workers must complete a project together: the more effort the junior worker exerts on the project, the less effort the senior needs to exert. The senior worker makes a suggestion about how much effort the junior worker should exert, and the latter can disobey, exposing herself to the chance of being punished. The experimental findings show that subjects in the role of senior workers systematically exploit junior workers; their attempts are more pronounced under asymmetric information.

Although the structure of our extortion game is similar, we depart in several aspects from both [22] and [23]. Given our conjecture that the peer’s behavior has an effect on whether or not an victim complies with extortive requests, we allow for the presence of multiple potential victims of extortion and analyze their interaction and the process of information sharing between them. Furthermore, since we are interested in studying situations where extortion might (or might not) emerge endogenously, our design contemplates the subjects’ possibility to self-select into the role of extorter or victim. Finally, we introduce a source of heterogeneity across subjects: victims can be extorted over sums they have earned, and not on a lump sum, fixed endowment; these sums may differ across victims.

Games reproducing appropriation from a common-pool resource ground on the seminal works by [24, 25] where a group of players takes part into a non-cooperative game in which each player makes an appropriative decision: in particular, players allocate an endowment between a common pool resource and a private alternative. The theoretical prediction is over-appropriation, in the same extent under-provision is expected in voluntary contribution mechanism game. As our design deals with the consequences of symmetric vs. asymmetric endowment of resources between the extorter and his victims, we are particularly interested in [25]’s result showing that, while symmetric provision and appropriation games produce comparable behaviors, asymmetry leads to significantly higher appropriation than in a payoff-equivalent provision game. The reason for this result is that second-movers generally react by reciprocating previous altruist or selfish behavior: when the opponents’ decision is observable, subjects with stronger power appear to be more sensitive towards opponents’ selfish choices and react by destroying surplus in order to sanction a level of appropriation they judge as “unfair”.

Similarly to our paper, the “power-to-take” game [26–27] can be used to capture agency situations where a principal decides on an incentives’ scheme for an agent, and the agent’s effort can be crowded out, thus reducing both his own and the principal’s payoff. Like ultimatum games, power-to-take games study dyadic interactions where either the request or the offer (respectively) turn out to be motivated by reasons that account for “profit maximizing” (proposers do not want their request or offer to be rejected), but also for “nonprofit-maximizing” behavior, i.e., are driven by fairness and inequality aversion considerations. In a related vein, [28] and [29] show that, when couples of negotiators interact with one another repeatedly and anonymously, the bargainers are little like those depicted by rational economic models: they offer too much, they reject offers that they should have accepted, and emotions rather than simple profits seem to have important effects on their behavior. Similarly, this paper investigates both economic and non-economic motivations underlying extortive requests and their compliance, and–among the latter–in addition to investigating fairness perception of extortive requests, it also accounts for peer effects as a source of compliance behavior among victims.

## Materials and methods

### Extortion game experiment: Design and procedure

Participants are divided in groups of three subjects, where one subject is assigned to the role A and the remaining two subjects are assigned to the role B. In the Instructions (see S1 File), we avoided the use of any loaded terms and referred to the players only with labels “A” and “B”, being A the potential extorter and B the potential victim of extortion. The presence of two subjects in the B role is motivated by the willingness to explore both the extorter consistency in behavior across multiple victims and peer effects on Bs behavior when receiving a feedback on the other B’s choice during the bargain process.

Subjects in each group interact for T = 10 periods. Each period is made of four stages (see below for a detailed description) and the composition of the group remains the same for all the ten periods of interaction (“partner protocol”).

The features of interaction that are common across treatments are the following: in each round, subjects of type B earn their endowment through a real effort task consisting of general knowledge multiple choice questions (see a sample of these questions in S1 File). Subjects of type A can make a request over each of the two subjects of type B’s earnings: they receive information on each B’s earned tokens and are asked how many tokens they want to take from them Bs are informed about A’s request and decide how much of her own earnings to give to A. If B rejects, then A has the possibility to punish B (see details below).

Bs earnings depends on both the correctness of their answer and the quickness of their answers. This choice has been made to represent a task where earnings depend on two types of effort: the former is related to the effort needed to pick the correct answer and requires ability but also concentration (we label it “quality effort”), the latter is captured by being rapid in reading/answering to questions (we label it “quantity effort”). Subjects receive the payment for quantity effort also in case their answer is wrong. We choose to use a task which requires both quality and quantity effort to explore whether any of them are influenced by the possibility of extortive request and whether they are crowded out if a subject is victim of appropriation.

Punishment consists in reducing Bs’ possibility to earn money in the next round: this occurs by eliminating from one to three questions in the next round set of questions B can answer to make money. Please note that the time is fixed for each question (and equal to 25 seconds): eliminating one question has the consequence of eliminating the whole slot of 25 seconds assigned to that question. Thus, in case of punishment, not only the subject loses the chance to get the prize for the correct answer, but also can save none of the 25 seconds allocated to that question. The prediction on the effect of punishment on earnings is then straightforward: fewer questions, fewer seconds available, less chance to answer correctly and fewer seconds to be saved. Punishment is costly for B, who can earn fewer tokens, but also for A: on one side, A pays a constant cost for each question that is eliminated. On the other, punishment reduces A’s chances of further appropriation: the less B earns, the less A can appropriate. There is no formal restriction on the punishment target (in principle, also compliant Bs can be punished, as in standard decentralized games with punishment *à la* [17]). However, we introduce a technology of punishment that is different from the more standard [17]’s one where punishment points reduce punishees’ earnings proportionally and can be assigned by the punisher at an increasing cost. The reason is that we are interested in representing a situation where the consequences of punishment are not limited to the period where punishment occurs, but affect subsequent interactions and influence also future earnings of both A and B. The tenth period of interaction crucially differs from periods 1 to 9 because subjects cannot punish, since there is no possibility to reduce the number of questions in the next period.

At the end of the experiment, subjects are asked to answer a post-experimental questionnaire aimed at collecting their demographic information, the intensity of the emotions they experienced during the experiment, and the level of request (as percentage of earnings) they judged as fair (see S1 File). This last question provides a benchmark to understand both how victims react in terms of bargaining in presence of requests higher than the “fair” ones, and how extorters respond to lower offers. Subjects were informed they would receive a final questionnaire after the end of the experiment, but they were not made aware about the specific content of the questions in order to avoid possible effects on their behavior during the experiment.

#### Treatments

In this section, a description of the four treatments of the “Extortion game” is provided. See Table 1 for an overview of the treatments.

**Table 1:** Treatments.

	ROLE ASSIGMENT	ENDOWMENT	PUNISHMENT	FEEDBACK ABOUT THE OTHER B’s COMPLIANCE
**T1: BASELINE**	Random	Symmetric	Yes	No
**T2: ROLE CHOICE**	Preference	Symmetric	Yes	No
**T3: ASYMMETRIC ENDOWMENT**	Preference	Asymmetric	Yes	No
**T4: FEEDBACK**	Preference	Asymmetric	Yes	Yes

*Baseline (Treatment 1)*. Subjects in each group interact for ten periods or rounds that are composed by four stages each. In the first stage, all the subjects earn their endowment by taking part in a real-effort task consisting of three general knowledge multiple-choice questions with four possible answers (where only one is correct). They have 25 seconds to answer each question. Earnings are expressed in tokens and depend on the number of correct answers (40 tokens for each correct answer) and on how fast they are in answering the questions (each second saved is worth one token). If the answer is wrong, the subjects do not receive the 40 tokens prize but can still save seconds and accumulate tokens: picking the answer at random could be a strategy for subjects who are not clever in this type of task since it allows maximizing at least the number of tokens they can earn by saving seconds. A has the chance to earn her endowment in the same way.

In the second stage, A receives a feedback on the amount π_B_ earned by each B in her group and decides how much to appropriate (*r*_*A*_) out of *π*_*B*_: also zero and the whole earning π_B_ are possible choices. The ratio *r*_*A*_*/ π*_*B*_ captures A’s proportional request (when proportion is set with respect to B’s earnings); the comparison between *r*_*A*_*/ π*_*B*_ and *r*_*A*_*/ π*_*-B*_ could be used as a proxy for A’s consistency in treating the two potential victims.

In the third stage, B receives A’s request and decides how much to give (*g*_*B*_) out of *r*_*A*_: also zero and the whole request *r*_*A*_ are possible choices. The ratio *g*_*B*_*/r*_*A*_ captures the degree of B’s compliance. Alternatively, the level of compliance can be defined as a percentage of earnings instead of as a percentage of the request (*g*_*B*_*/ π*_*B*_).

In the fourth stage, A receives a feedback on *g*_*B*_ and has the chance to punish each B in her group, by eliminating one or more (up to all the three) questions. A has three options: eliminating one question at the cost of 10 tokens, eliminating two questions at the cost of 20 tokens, eliminating three questions at the cost of 30 tokens (. In other words, A can choose between 1, 2 or 3 punishment points. The four stages are summarized in Table 2.

**Table 2:** Summary of the four stages.

**Stage 1**	Real effort task: general knowledge multiple choice questionnaire
**Stage 2**	A receives a feedback on Bs’ earnings and makes the extortive request
**Stage 3**	Bs receive A’s request and choose their level of compliance
**Stage 4**	A receives a feedback on Bs’ compliance and decide whether/how much to punish

At the end of each round, each subject receives a detailed feedback on the composition of his earnings in that round. In case of subjects As, earnings are made by the tokens earned from correct answers, plus the tokens earned by saving seconds, plus the eventual tokens extorted. In case of subjects Bs, earnings are made by the tokens earned from correct answers, plus the tokens earned by saving seconds, minus the eventual tokens extorted.

*Role choice (Treatment 2)*. This treatment differs from the Baseline in the fact that the roles of A or B are assigned after subjects express their preference for assuming the role of A by answering to the following statement: “Please express your preference for assuming the “A” role instead of the “B” role from 1 (no desire at all) to 10 (very strong desire)”. The subjects make this decision after being aware of all the relevant features of both roles, and before starting the ten rounds of interaction. In case of ties in preference levels among two or more subjects, the computer assign the “A” role at random. The rationale behind this treatment is understanding whether appropriative behavior is the result of idiosyncratic characteristics that cause people to self-select into the role of extorter, or on the contrary whether it is the context that shapes behavior, being any person that can exploit another person potentially able to take advantage of her position.

*Role choice + asymmetric endowment (Treatment 3)*. This treatment differs from the previous in the fact that *only* subjects of type B can earn their money by answering questions; subjects of type A can make money only by appropriating subjects B’s earnings. In this treatment, subjects As’ earnings are made only by the eventual tokens extorted, minus the eventual cost of punishment. With this treatment, we aim at investigating whether another contextual factor, namely the unevenness in the distribution of the resources, is able to affect appropriative behavior through the possibility for the subject to find a self-justification for behaving badly (“I have a lower chance than my peers to make money, thus I am allowed to take part of their earnings”).

*Feedback (Treatment 4)*. This treatment differs from Treatment 3 in the fact that subjects of type B receive feedback on the level of compliance of the other *B* in their group in the previous period. The feedback is reported as follows: “In the previous round, the other B subject in your group has given *X* tokens out of the *Y* tokens A has requested”.

#### Participants and procedures

The experiments have been conducted at the CESARE lab of LUISS University in Rome, Italy. Subjects were recruited *via* ORSEE [30]. The experiment was programmed by using the z-tree platform [31]. We ran 8 computerized sessions, between June 2013 and July 2013, with a total of 171 participants (45 subjects in Treatments 1, 2 and 4; 36 subjects in Treatment 3). Participants were undergraduate students (63.2% from Economics), with 57.3% males. We employed a between-subjects design: no individual participated in more than one session. In each session, the participants were paid a 2€ show-up fee, plus their earnings from the experiment. At the beginning of each session, participants were welcomed and, once all of them were seated, the instructions were handed to them in written form before being read aloud by one experimenter. All subjects completed a final questionnaire containing demographic information, a statement about the level of request (as percentage of earnings) they judged as “fair”, and a set of 16 questions where they had to self-report (in a 1–7 scale) the intensity of the emotions they felt during the experiment. The sessions took approximately 45 minutes, with earnings ranging between 2€ and 19€. All participants provided written informed consent. The study has been approved by the *National Research Council* of Italy Ethics committee.

## Results

### Descriptive statistics

Descriptive statistics on levels of earnings, effort (qualitative effort corresponds to the amount of tokens earned because of correct answers, while quantitative effort corresponds to the amount of tokens earned because of seconds saved), requests (expressed as percentage of earnings) and compliance (expressed as percentage of the request and as percentage of earnings, respectively) in the four treatments are summarized in Table 3.

**Table 3:** Descriptive statistics on earnings, effort, requests and compliance.

	1	2	3	4
Variable (averages)	Baseline	Role choice	Role choice + As End	Feedback
**Bs’ earnings**	88.9	97.3	81.4	86.4
**Bs’ qualitative effort**	58.3	63.5	60.5	64.7
**Bs’ quantitative effort**	30.7	33.8	20.9	21.7
**As’ request level = A’s Request/B’s earnings**	35.6	31.9	45.7	48.0
**Bs’ compliance 1 = A’s appropriation/** **A’s request**	28.6	34.3	33.1	32.9
**Compliance 2 = A’s appropriation/** **B’s earnings**	0.11	0.12	0.17	0.18

Mann-Whitney rank-sum tests on significance differences are reported in Table 4: the first column refers to subjects Bs’ earnings (we focus on subjects Bs’ earnings because we are interested in relating them to requests and compliance), the second and third to effort, the fourth to requests, the fifth and the sixth to compliance (defined as percentage of requests, or as percentage of earnings, respectively) and the seventh to the preference for A role.

**Table 4:** Significance levels of difference in earnings, requests, compliance and preference for A role across treatments.

Treatments	Mann-Whitney Rank-sum test						
	Bs’ Earnings	Bs’ qualitative effort	Bs’ quantitative effort	Request	Compliance	Compliance 2	Preference for A role
1 (Baseline) vs 2 (Role choice)	**p < .001**	**p = .004**	**p < .001**	**p = .065**	**p = .180**	**p = .404**	**-**
	z = 11.285	z = -2.881	z = 4.404	z = -2.19	z = 1.511	z = .835	
2 (Role choice) vs 3 (Role choice+As End)	**p < .001**	**p = .031**	**p < .001**	**p < .001**	**p = .837**	**p < .001**	**p < .001**
	z = 10.68	z = -2.151	z = -13.031	z = -13.3	z = -0.123	z = 4.952	z = -7.564
3 (Role choice+As End) vs 4 (Feedback)	**p < .001**	**p < .001**	**p < .001**	**p = .541**	**p = .312**	**p = .957**	**p = .687**
	z = 12.420	z = 5.793	z = 6.631	z = 0.780	z = -1.144	z = .053	z = -0.402

Interestingly, subjects Bs’ earnings are significantly different across treatments and vary between 81.4 and 97.3 tokens. Please note that subjects B answer exactly the same questions across treatments, thus any average difference in earnings, if present, should depend on the treatment itself. In particular, when subjects have expressed a preference for the role to play, earnings are significantly higher, corresponding to both a higher qualitative effort (tokens earned because the answer is correct) and a higher quantitative effort (tokens earned because subjects respond fast and save more seconds). The result is driven by the fact that subjects As have now the chance to self-select in the role of extorters and thus earn money (also) by taking Bs’ earnings. When choosing their role, subjects seem to be well aware of their capabilities and to anticipate their performance in the task quite precisely: the less clever they are, the more they choose to play the role of extorter. We find a negative and significant correlation between subjects’ preference for A’s role and measures of their performance in the task, i.e. the quantity effort, as measured by the amount of seconds they save when answering to the questionnaire—that are converted into tokens in order to reward quick answers—and quality effort—that capture their competence and attention in answering correctly (Spearman correlation test, with coef. = -.118, p < .001, and coef. = -.317, p < .001). When the endowment becomes asymmetric (treatment 3), Bs’ earnings drop: Bs probably anticipate they will receive higher requests and will have to redistribute earnings, and they put lower qualitative (the tokens earned because the answer is correct reduce from 63.5 to 60.6) and quantitative effort (they save on average less seconds, from 33.8 to 20.8). Finally, when feedback is available, subjects Bs put higher effort of both types and thus earn more: we explain this result relying on the fact that subjects know they will be compared to peers (peers will know about the requests they receive and can indirectly derive information on their earnings and performance, as they can do about peers), attaching some value to the fact of appearing clever and so putting higher effort in the task. This holds both in terms of qualitative than quantitative effort. An objection to this analysis can relate to the fact that Bs’ earnings can be affected by punishment, that reduces the number of questions Bs can answer. However, as we will see below, we do not find any significant difference in the use of punishment across treatments.

Overall, the level of the requests ranges between 31.9% and 48% of earnings, with major variations across treatments. Requests significantly increase when the endowment becomes asymmetric, since subjects As probably feel justified to make higher requests because they do not have the chance to earn any money by themselves. There is a slight increase in requests also when the role choice is endogenous (with respect to the Baseline), which is likely due to the fact that subjects who are more prone to make requests self-select into A’s role.

We now move to test our hypothesis that compliance to extortive request is conditional to the behavior of other victims. When we define compliance as the ratio between the tokens offered and the tokens requested (“compliance 1”), we find no differences in the levels of compliance between treatments 1, 2, 3, and 4. We conducted a power analysis to verify whether our sample size is large enough to be able to conclude that the differences in Bs’ compliance behavior between the Baseline and treatments are not significant, and can interpret the null effects on B’s compliance by observing that, if there should be any effects that we might have failed to detect, these effects are most likely very small. We report test power for exemplary effect sizes (large, medium and small effects, as outlined in [32]). We assume power .8 and an alpha level of 0.05; we also account for a repeated measures design (ten rounds) and follow [33]’s conservative approach on the prediction of the correlation assuming a covariance pattern with autocorrelation of 0.5. If we anticipate that the effect of our treatments is small, we posit an effective size index dE equal to 0.28 (size index d = 0.2 adjusted for the assumed correlation 0.5) and obtain a required sample size of 370 per sample. If we anticipate that the effect of our treatments is medium, we posit an effective size index dE equal to 0.70 (size index d = 0.5 adjusted for the assumed correlation 0.5) and obtain a required sample size of 33 per sample. If we anticipate that the effect of our treatments is large, we posit an effective size index dE equal to 1.13 (size index d = 0.8 adjusted for the assumed correlation 0.5) and obtain a required sample size of 12 per sample.

Since our sample size in the three treatments is 300, 240 and 300, respectively (see the last column of Table 1), we conclude that our study has sufficient power for all but very small effects. Significance levels of difference in compliance are reported in the fifth column of Table 4 above.

Yet, subjects Bs’ levels of compliance positively and significantly depend on the specific feedback they receive on the amount of tokens their peer has been requested and gave (Spearman correlation test, with coef. = .347 and p < .001). Since the average compliance level across treatments is around 30%, an immediate figure to illustrate this behavior is showing that subjects who learnt that the other B in the group had offered 30% of requested tokens or more reacted by offering on average 47% of requested tokens. On the contrary, subjects who learnt that the other *B* in the group had offered less than 30% of the requested tokens reacted by offering on average 23%. Both compliance levels (in percentages) are significantly different from the one observed in Treatment 3, that differs only for the fact that after each interaction B receives a feedback on the other B’s previous degree of compliance. Victims’ compliance is therefore conditional to the behavior of other victims, supporting our hypothesis. Consistent with the fact that there is no difference in the set of actions and information for subjects A, the average request (48.0%) is not significantly different from the one in Treatment 3 (Wilcoxon rank-sum test on individual averages, with p = 0.541, two-tailed test).

Column 6 refers to our second definition of compliance (“compliance 2”, i.e. the tokens offered as a percentage of the earnings). The only difference emerging with respect to the previous definition of compliance regards the fact that we observe a significant difference in compliance between treatments 2 and 3: compliance as a percentage of earnings significantly increases when the endowment becomes asymmetric. As emphasized above when commenting on request levels across treatments, subjects As seem to compensate the fact they have no chance to earn her earnings by asking more, and consequently Bs give more. With the exception of the Baseline (Treatment 1), the attribution of experimental role is not random, but based on a preference for playing the role of A instead of B expressed by all subjects at the beginning of the experiment. As anticipated above, this preference is expressed in a scale from 1 to 10, where 10 is the maximum willingness to assume the A role: the computer assigns the A role to the subjects in the session who expressed the highest preference for it and implements a random draw in case of ties. This is a non-incentivized question and there is no auction to obtain the role of A. Table 5 summarizes the average level of preference expressed by subjects in the four treatments.

**Table 5:** Average preference for A role.

	1	2	3	4
	Baseline	Role choice	Role choice + As End	Feedback
Average preference for A role	-	6.91	5.04	5.15

Not all subjects state the maximum willingness to be in the role of participant A. This result is interesting since the choice of playing as A satisfies both the goal of selfish types that can extort others and that of non-selfish types that can prevent extortion of themselves and others.

Not surprisingly, the preference for A’s role is significantly lower when As have no way to earn money other than taking tokens from their Bs (what we call “asymmetric endowment”). Indeed, the average preference expressed in Treatment 2 (that equals 6.91), where As also have the questionnaire task as source of earnings, is significantly higher (although at the 6.5 percent level) than the average preference expressed in all other treatments. This result make sense since in Treatment 2 subjects are aware they have two sources of earnings (their performance in the questionnaire and the earnings from extortion) instead of one only (the earnings from extortion) as in the other treatments.

As emphasized above, choosing the role of A instead of B determines a significant drop in A’s average request, compared to when the role of A is assigned randomly. Subjects who self-select into the role of extorter end up into making significantly lower requests: on average, the levels of percentage request are 35.6% in Treatment 1 (“Baseline”) and 31.9% in Treatment 2 (“Role choice”) (Wilcoxon rank-sum test on individual averages, with p = 0.065, two-tailed test; see Table 4). A possible rationale for this finding is the following: when choosing the role of A, individuals feel responsible towards their peers and make moderate requests; when randomly assigned to the role of A, they can shift the responsibility of acting greedily to external factors and find a justification for high requests. This result is consistent with the experimental evidence in [34], showing that a shift of responsibility to an external authority diminishes internal impulses towards prosocial behavior. This finding is also in line with previous evidence showing that subjects might prefer to shift the responsibility to take a decision that affects others’ well-being, see for instance [35]’s paper on delegation. Whereas in the context of delegation, the delegee might act selfishly because she feels she is just carrying out orders, in our case participants who are assigned the role of A extort because playing according to the role they received. In sum, the preference for the role of A seems to be influenced by a set of factors moving in opposite directions: the expectation to earn more (that determines a decrease in the preference when As have no endowment and also an increase in preference for subjects who were less good in the task), but also the desire to have the control and behave fairer (since As who chose their role request less than As who were assigned to that role).

Finally, Table 6 provides a summary of the number of punishment points that subjects Bs receive in all the four treatments.

**Table 6:** Punishment points received across treatments.

	1	2	3	4
# punishment points received	Baseline	Role choice	Role choice + As End	Feedback
0	190	162	196	201
1	68	56	57	57
2	15	15	25	25
3	27	7	19	17

In general, punishment is used in less than 30% of cases. Subjects Bs who are punished experience the elimination of one, two or three questions in the following round with the consequent impossibility to earn money by answering the question(s). There is no difference across Treatments 1, 2, 3 and 4 in frequency and intensity (one, two or three questions eliminated) of punishment: overall, the number of punishment points decreases as interaction goes on, because significantly fewer subjects inflict punishment (Wilcoxon rank-sum test on individual averages, with p = 0.033, one-tailed test). The use of punishment of low intensity (one question eliminated) is the most chosen strategy by extorters in all the treatments: it represents the 61.3% of punishment points whereas two or three questions are eliminated in 20.6% and 18% of cases respectively.

### Regression analysis

The following regressions provide a deeper analysis of the determinants of A’s request and of B’s compliance, received punishment and performance in the task.

### A’s request

Table 7 illustrates the determinants of the request level under a set of different specifications. In regressions reported in columns 1–7 errors are clustered at the subject level, but results hold when error are clustered at the group level (column 8). The level of the request significantly and positively depends on (I) the victim’s earnings, (II) the request made to the other victim, (III) the presence of an asymmetric endowment (i.e. the extorter asks for more when extortion is the only source of income). The level of the requests depends significantly but negatively on (IV) the other victim’s earnings, (V) the period of interaction (the longer the interaction, the lower the request), (VI) the choice of the role (when the subject can self-select into the role of extorter he turns to behave less greedily). These results hold when excluding the last period of interaction—when punishment is not possible—(column 2), and when controlling for: treatments (column 3), the level of request that is perceived as fair (that is significant and positive) (column 4), the level of compliance in the previous period (column 5), the level of quality and quantity effort of the victim in the same period (column 6), and demographic characteristics (column 7). We also observe a positive relationship between the level of the request and the degree of compliance in the previous period: the extorter seems to ask more to the subjects who has been more compliant in the past. Furthermore, there is consistency between the level of request the extorter considers “fair” and his actual request. We found neither gender effect, nor any role for major and age.

**Table 7:** Determinants of A’s request.

	(1)	(2)	(3)	(4)	(5)	(6)	(7)	(8)
A’s request to *Bi*		no last period	treatments	perceived fairness	lagged compliance	current effort	Dem. controls	group cluster
*Bi'*s earnings	0.414***	0.418***	0.416***	0.418***	0.397***	0.412***	0.415***	0.414***
	[0.028]	[0.030]	[0.028]	[0.028]	[0.028]	[0.028]	[0.028]	[0.028]
*Bj'*s earnings	-0.264***	-0.267***	-0.252***	-0.237***	-0.241***	-0.240***	-0.253***	-0.264***
	[0.025]	[0.025]	[0.024]	[0.025]	[0.026]	[0.028]	[0.025]	[0.025]
Request to *Bi*	0.670***	0.673***	0.648***	0.604***	0.624***	0.622***	0.640***	0.670***
	[0.049]	[0.049]	[0.047]	[0.053]	[0.051]	[0.057]	[0.048]	[0.049]
Period	-0.414*	-0.619**	-0.434*	-0.457*	-0.219	-0.340	-0.432*	-0.414*
	[0.244]	[0.279]	[0.245]	[0.247]	[0.301]	[0.302]	[0.245]	[0.242]
Asym. endowment			8.422**	4.736**			5.057**	
			[3.395]	[2.219]			[2.282]	
Role choice			-6.186*					
			[3.400]					
Feedback			0.405					
			[2.869]					
Fair request				0.223***				
				[0.073]				
Bi's compliance in t-1					0.199***			
					[0.065]			
Quantity effort						-0.084		
						[0.067]		
Quality effort						-0.010		
						[0.028]		
Gender							-1.827	
							[2.124]	
Age							0.266	
							[0.180]	
Major							3.142	
							[2.143]	
Constant	1.171	1.618	0.841	-9.128***	-1.725	2.219	-8.865*	1.171
	[1.660]	[1.928]	[2.024]	[3.334]	[1.902]	[2.254]	[4.978]	[1.593]
Observations	570	513	570	570	513	513	570	570
Number of subject	57	57	57	57	57	57	57	57

GLS regression. Tobit estimation gives very similar results. Robust standard errors in brackets.

*** p<0.01

** p<0.05

* p<0.1

In sum, we find evidence suggesting that A’s request is proportional to subject B’s earnings, since the coefficient is positive and significant. Furthermore, subject A makes similar requests to both Bs, showing a high degree of consistency in the behavior towards his potential victims.

### B’s compliance

Table 8 summarizes the determinants of the increase in percentage compliance across all the treatments.

**Table 8:** Determinants of B’s increase in compliance in all treatments.

*Bi*'s % compliance in t—*Bi'*s % compliance in t-1	(1)	(2)	(3)	(4)	(5)	(6)
		treatments	perceived fairness	effort	dem. controls	group cluster
Punishment received by *Bi*	0.058**	0.058**	0.058**	0.022	0.059**	0.058**
	[0.024]	[0.025]	[0.025]	[0.026]	[0.025]	[0.027]
Role choice		0.004				
		[0.016]				
Asym. Endowment		-0.008				
		[0.015]				
Feedback		-0.003				
		[0.014]				
Fair request			0.000***			
			[0.000]			
Quantity effort				-0.002***		
				[0.001]		
Quality effort				-0.001		
				[0.000]		
Gender					-0.015	
					[0.010]	
Age					0.002	
					[0.004]	
Major					-0.008	
					[0.011]	
Constant	-0.053***	-0.050***	-0.056***	0.050	-0. 081	-0.053***
	[0.010]	[0.012]	[0.011]	[0.032]	[0.089]	[0.011]
Observations	811	811	811	811	811	811
Number of subject	109	109	109	109	109	109

GLS regression. Tobit estimation gives very similar results. Robust standard errors in brackets.

*** p<0.01

** p<0.05, * p<0.1

Subjects Bs’ increase of compliance in time depends significantly and positively on the intensity of punishment received in the previous period, with the exception of column 4 where we control for current effort. The regression confirms that treatments play no role (column 2). Punishment is effective in sustaining compliance, and this result is robust when adding the level of request perceived as fair (column 3) and demographic variables as controls (column 4). As above, clustering errors at the group level does not alter the result (column 5). Despite the differences in the punishment technology, this result confirms the findings about altruistic punishment in public good games [17] as well as in previous extortion games [22].

Notably, the level of compliance drops consistently in the last period, when punishment is not possible: the average level of compliance in the 10^th^ round is 17.5%.

While Table 8 refers to the increase in compliance from a period to the next one, Table 9 considers compliance in a given period and provides a deeper investigation of the role of feedback in shaping compliance in Treatment 4.

**Table 9:** Determinants of B’s compliance in Treatment 4.

	(1)	(2)	(3)	(4)	(5)	(6)
*Bi*'s compliance in t	punishment only	request	perceived fairness	info	controls	group cluster
Request received by *Bi* in t-1		0.128	0.128	0.119	0.116	0.119
		[0.078]	[0.078]	[0.080]	[0.080]	[0.080]
*Bi*'s earnings	0.174***	0.111***	0.111***	0.119***	0.119***	0.119***
	[0.039]	[0.042]	[0.042]	[0.041]	[0.041]	[0.041]
*Bj*'s compliance in t-1				0.190***	0.186***	0.190***
				[0.064]	[0.066]	[0.064]
Punishment received by *Bi*	0.246	0.212	0.212	0.548	0.558	0.548
	[0.935]	[0.808]	[0.808]	[0.812]	[0.825]	[0.812]
Period		-0.001	-0.001	-0.070	-0.070	-0.070
		[0.443]	[0.443]	[0.418]	[0.428]	[0.418]
Gender					-3.377	
					[7.203]	
Age					-0.354	
					[1.132]	
Major					0.665	
					[6.909]	
Fair request		-0.016	-0.016			
		[0.184]	[0.184]			
Constant	2.962	3.348	3.348	-0.460	9.500	-0.460
	[2.214]	[7.615]	[7.615]	[4.186]	[29.790]	[4.186]
Observations	120	120	120	120	120	120
Number of subject	15	15	15	15	15	15

GLS regression. Tobit estimation gives very similar results. Robust standard errors in brackets.

*** p<0.01, ** p<0.05, * p<0.1

When the information on the other victim’s compliance is available, as happens in Treatment 4, each subject B adapts her own level on compliance on it. Interestingly, punishment plays no role. When including feedback in the set of regressors (column 4), the reason becomes clear: punishment is not effective in this treatment because comparatively less salient than the feedback on the other peer’s behavior. This result is robust when controlling for the request received (columns 2–6), for the level of request perceived as fair (column 3), for demographic characteristics (column 5). Clustering at the group level (column 6) does not change the results.

A careful inspection of the data reveals that, while the difference in the level of percentage compliance between the two Bs is 42.03% in the first period, from the second period on it drops to 8% and remains stable across periods, with the exception of the last period, when it rises again and reaches 17.89%. This analysis provides further evidence that victims’ compliance is conditional to the behavior of other victims.

### Punishment

Table 10 investigates the determinants of the amount of punishment points that a subject of type B receives.

**Table 10:** Determinants of B’s received punishment points.

	(1)	(2)	(3)	(4)	(5)
Punishment received by *Bi*		no last period	treatments	controls	group cluster
Request received by *Bi* in t-1	0.006***	0.008***	0.008***	0.008***	0.008***
	[0.002]	[0.002]	[0.002]	[0.002]	[0.002]
*Bi*'s compliance in t-1	-0.020***	-0.024***	-0.024***	-0.024***	-0.024***
	[0.003]	[0.003]	[0.003]	[0.003]	[0.004]
Period	-0.045***	-0.010	-0.010	-0.010	-0.010
	[0.007]	[0.009]	[0.009]	[0.009]	[0.011]
Role choice			-0.095		
			[0.153]		
Asym. Endowment			0.101		
			[0.115]		
Feedback			-0.014		
			[0.129]		
Gender				0.084	
				[0.091]	
Age				0.005	
				[0.034]	
Major				0.076	
				[0.112]	
Constant	0.782***	0.659***	0.683***	0.446	0.659***
	[0.080]	[0.076]	[0.132]	[0.822]	[0.091]
Observations	1,138	1,024	1,024	1,024	1,024
Number of subject	114	114	114	114	114

GLS regression. Tobit estimation gives very similar results. Robust standard errors in brackets.

*** p<0.01, ** p<0.05,* p<0.1

Unsurprisingly, punishment depends significantly and negatively on the previous degree of compliance: the more a subject B meets A’s requests, the lower the number of punishment points he receives. Furthermore, punishment depends significantly and positively on A’s request. Column 1 shows a negative relationship with time: it looks as if, as the interaction goes on, punishment declines. However, if we exclude the last period of interaction, this decrease in time of punishment is no more significant, suggesting that such an effect was driven by the last period. The regressions in columns 2–5 exclude the last period of interaction and show that results hold when controlling for treatments (column 3), for demographic features (column 4), and if clustering at the group level (column 5).

Further analyses on the effects of extortion on Bs’ effort and on self-reported measures of the emotions experienced during the experiment are reported in the S1 File.

## Discussion and conclusions

This paper presents an investigation of both the extorter’s and the victim’s behavior in an “extortion game” with multiple victims. Our design contemplates the presence of multiple victims, that allows us to be the first at testing the effect of peers’ behavior on individuals’ compliance with appropriative requests.

Additionally, our design enables to manipulate the symmetry in the distribution of initial resources between the extorter and the victims, and subjects’ possibility to self-select into the role of extorter or victim. These new features help a deeper comprehension of the determinants of the interaction among extorters and victims of extortion.

Our results show that in general extorted sums are rather small about 10% - 15% of the victim’s earnings. Requests are proportional to the victim’s profits and similar across victims. Moreover, the extorter who self-selects into this role makes significantly lower requests, compared to the extorter who acquires the role randomly. The same is true for the extorter who has his own source of earnings compared to the one that has extortion as the only source of income.

Moreover, our results show that that punishment is rare, but plays a crucial role in increasing compliance with extortive requests. This result differs from [22] findings, where punishment is massive and increasing in time: the reason is likely to depend on the fact that this form of punishment consists of reducing also the punisher’s possibilities of earnings. Furthermore, punishment costs increase in punishment intensity instead of being fixed. Yet our results show that in addition to punishment victims are also sensitive to other factors. In particular, fairness perceptions play an important role: the requests made by extorters are positively correlated with the request they perceive as fair. In addition, victims who are required to pay what they consider to be an unfair amount show lower levels of compliance and experience higher intensities of emotions, such as anger and irritation, than when the request is perceived as fair.

Finally, in agreement with our hypothesis, we find that when the information on peers’ behavior is available, compliance is affected by conformism among victims rather than by punishment. Victims tune their compliance on the behavior of their peers, showing that conformity to others’ conduct represents a strong and robust drive of human behavior [36–38] and that social norms influence individuals’ conduct also in situations that are not beneficial for the society.

In sum, our findings show that, besides punishment, conforming to peers’ level of compliance is a key driver of individuals’ obedience to the requests of an authority, even in contexts that are socially undesirable or harmful. These may be considered as factors sufficient to induce obedience independently from the content of request and possibly from the specific nature of the authority.

## Supporting information

S1 File Supporting information for Peer effects on compliance with extortive requests. (DOCX)

S1 Dataset (XLSX)
