# Supplementary material for: Peer effects on compliance with extortive requests
Source: PLoS One. 2020 Apr 24;15(4):e0231879. doi: 10.1371/journal.pone.0231879 (PMC7182209; doi:10.1371/journal.pone.0231879)
Supplement: S1 File — (DOCX) [file pone.0231879.s001.docx]

**Supporting Information for Peer effects on compliance with extortive requests**

***Determinants of Bs’ effort***

What is the effect of successful extortions and punishment of non-compliant victims on the overall economic activity of its victims? Is there any crowding-out effect? The following regressions show that the answer is no with regards to extortion *per se*, but yes with regards to punishment.

**Table S1. Determinants of B’s “quantity” effort in all treatments**

|  | (1) | (2) | (3) | (4) | (5) | (6) |
| --- | --- | --- | --- | --- | --- | --- |
| *Bi*'s “quantity” effort |  | treatments | period | no last period | controls | group cluster |
|  |  |  |  |  |  |  |
| *Bi*'s compliance in t-1 | 0.152*** | 0.168*** | 0.152*** | 0.181*** | 0.151*** | 0.152*** |
|  | [0.045] | [0.039] | [0.044] | [0.045] | [0.046] | [0.050] |
| Punishment received by *Bi* | -0.697 | -2.031*** | -0.835 | -1.094 | -0.696 | -0.697 |
|  | [0.686] | [0.718] | [0.675] | [0.747] | [0.679] | [0.737] |
| Role choice |  | 2.088 |  |  |  |  |
|  |  | [2.227] |  |  |  |  |
| Asym. Endowment |  | -12.962*** |  |  |  |  |
|  |  | [1.708] |  |  |  |  |
| Feedback |  | 0.519 |  |  |  |  |
|  |  | [1.540] |  |  |  |  |
| Period |  |  | 0.834*** | 0.670*** |  |  |
|  |  |  | [0.125] | [0.182] |  |  |
| Gender |  |  |  |  | -0.917 |  |
|  |  |  |  |  | [1.806] |  |
| Age |  |  |  |  | -0.165 |  |
|  |  |  |  |  | [0.537] |  |
| Major |  |  |  |  | -3.166* |  |
|  |  |  |  |  | [1.909] |  |
| Constant | 23.523*** | 29.153*** | 18.582*** | 19.051*** | 29.660** | 23.523*** |
|  | [1.408] | [1.942] | [1.459] | [1.589] | [12.686] | [1.650] |
|  |  |  |  |  |  |  |
| Observations | 1,023 | 1,023 | 1,023 | 909 | 1,023 | 1,023 |
| Number of subject | 114 | 114 | 114 | 114 | 114 | 114 |

*Note. GLS regression. Tobit estimation gives very similar results. Robust standard errors in brackets. *** p<0.01,*

*** p<0.05, * p<0.1*

**Table S2. Determinants of B’s “quality” effort in all treatments**

|  | (1) | (2) | (3) | (4) | (5) | (6) |
| --- | --- | --- | --- | --- | --- | --- |
| *Bi*'s “quantity” effort |  | treatments | period | no last period | controls | group cluster |
|  |  |  |  |  |  |  |
| *Bi*'s compliance in t-1 | 0.303*** | 0.293*** | 0.302*** | 0.346*** | 0.301*** | 0.303*** |
|  | [0.082] | [0.080] | [0.081] | [0.086] | [0.084] | [0.090] |
| Punishment received by *Bi* | -7.948*** | -7.898*** | -8.138*** | -8.529*** | -7.902*** | -7.948*** |
|  | [2.163] | [2.163] | [2.155] | [2.288] | [2.130] | [2.226] |
| Role choice |  | 4.222 |  |  |  |  |
|  |  | [3.872] |  |  |  |  |
| Asym. Endowment |  | -3.827 |  |  |  |  |
|  |  | [3.304] |  |  |  |  |
| Feedback |  | 3.567 |  |  |  |  |
|  |  | [3.631] |  |  |  |  |
| Period |  |  | 1.179*** | 1.376*** |  |  |
|  |  |  | [0.371] | [0.470] |  |  |
| Gender |  |  |  |  | -0.163 |  |
|  |  |  |  |  | [2.530] |  |
| Age |  |  |  |  | -0.442 |  |
|  |  |  |  |  | [0.846] |  |
| Major |  |  |  |  | -2.413 |  |
|  |  |  |  |  | [2.851] |  |
| Constant | 62.434*** | 60.504*** | 55.465*** | 54.216*** | 73.990*** | 62.434*** |
|  | [2.497] | [3.446] | [3.403] | [3.530] | [20.613] | [2.740] |
|  |  |  |  |  |  |  |
| Observations | 1,023 | 1,023 | 1,023 | 909 | 1,023 | 1,023 |
| Number of subject | 114 | 114 | 114 | 114 | 114 | 114 |

*Note. GLS regression. Tobit estimation gives very similar results. Robust standard errors in brackets. *** p<0.01,*

*** p<0.05, * p<0.1*

In each period, both types of effort are significantly and positively affected by the amount of tokens extorted in the previous period: the higher a subject’s level of compliance in the previous period, the more he works for answering either correctly and quickly. However, the amount of punishment received in the previous period crowds out quality effort. Furthermore, subjects increase both types of effort as interaction goes on, probably because they become more familiar with the task (column 3 in both tables). This effect holds also when excluding the last period of interaction (see column 4 in both tables). Results on compliance and time hold also when controlling for treatments (column 2), demographic features (column 5), and if clustering at the group level (column 6). On the contrary, the negative effect of punishment on quantity effort holds only when we control for treatments and depend in particular on the Asymmetric Endowment feature. In this case, we observe that subjects are slower in answering questions and this result is likely to be due to the significantly higher level of extortion that As exert when they have no alternative possibility to make money. As shown in Table S2, punishment always crowds out the number of correct answers no matter the specification: we interpret this result by accounting for the fact that answering correctly is more time-consuming and difficult for subjects. If extorted, they start answering at random without reading the question in order to find an easier way to make money.

***Fair request and emotions***

In the final questionnaire, we ask subjects in both A and B roles to assess the level of request (in percentage of earnings) they judge as “fair” (see [1] for research on subjects’ perception of fairness norms) and to express in a scale from 1 to 7 the intensity of a number of emotions they could experience during the experiment (happiness, guilt, shame, surprise, envy, anger, sadness, irritation, pride, gratitude, admiration, fear, blame, disappointment, grief, indignation). The list of emotions and the methodology used for measuring them is based on [2].

Table S3 summarizes descriptive statistics on the self-reported level of these emotions and the request judged as fair, considering subjects As and Bs separately.

**Table S3. Summary statistics on emotions and fair request (from the post-experimental questionnaire)**

|  |  |  |  |  |  |
| --- | --- | --- | --- | --- | --- |
|  | Subjects A | |  | Subjects B | |
| **Self-reported item** | **average** | **std. err** |  | **average** | **std. err** |
| Happiness | 2.70 | 0.070 |  | 3.21 | 0.053 |
| Guilt | 1.89 | 0.057 |  | 1.73 | 0.05 |
| Shame | 1.37 | 0.034 |  | 1.67 | 0.043 |
| Surprise | 3.28 | 0.068 |  | 3.75 | 0.053 |
| Envy | 2.47 | 0.073 |  | 1.97 | 0.05 |
| Anger | 3.22 | 0.068 |  | 3.01 | 0.045 |
| Sadness | 1.98 | 0.089 |  | 1.74 | 0.063 |
| Irritation | 3.91 | 0.083 |  | 3.47 | 0.066 |
| Pride | 3.61 | 0.089 |  | 3.01 | 0.061 |
| Gratitude | 3.01 | 0.076 |  | 2.52 | 0.056 |
| Admiration | 2.23 | 0.069 |  | 1.96 | 0.047 |
| Fear | 1.35 | 0.041 |  | 1.57 | 0.035 |
| Blame | 2.49 | 0.081 |  | 2.18 | 0.053 |
| Disappointment | 3.96 | 0.085 |  | 3.39 | 0.061 |
| Grief | 2.79 | 0.082 |  | 2.49 | 0.056 |
| Indignation | 2.60 | 0.082 |  | 2.07 | 0.052 |
|  |  |  |  |  |  |
| Fair request | 34.89 | 0.743 |  | 27.39 | 1.745 |
|  |  |  |  |  |  |

*Note: Emotions are reported in a 1-7 scale. Fair request corresponds to the percentage* *level of request*

*the subject considers “fair”.*

The overall average self-reported “fair request” is about 35% of earnings for subjects As, whereas subjects Bs reported a percentage request of 27% (the difference is significant, Wilcoxon rank-sum test on individual averages, with z= 14.462 and p = 0.0001, two-tailed test). Comparing across treatments, we find that the fair request is significantly higher for subjects Bs in the Baseline, who show an average percentage of 40.7% compared to 25.7% of subjects Bs in the other treatments (Wilcoxon rank-sum test on individual averages, with with z= 3.573 and p = 0.0004, two-tailed test): this could serve as confirmation that receiving the role of A at random and then behave consistently is judged less negatively in terms of responsibility also by subjects Bs. In fact, Bs tolerate a significantly higher request, and consider it as “fair” only in the treatment where As did not choose the role (i.e., the Baseline). Furthermore, for subjects Bs, compliance is significantly and positively correlated to the level of request judged as “fair” (coef. = 0.085, p=.0001).

For what concerns self-reported emotions, the emotions that prevail for subjects As are disappointment, irritation and pride, whereas for subjects Bs are surprise, irritation and disappointment. The correlation analysis allows to go more in depth and shows that irritation and anger are significantly and positively correlated with the distance between the average request received and the request judged as “fair” (coef. = 0.050, p=.039; coef. = 0.085, p=.0001, respectively). In general, disappointment and irritation are negatively and significantly correlated with profits (coef. = -0.212, p<.000; coef. = -0.169, p=.0001, respectively). For subjects As, happiness and gratitude increase in Bs’ compliance (coef. = 0.262, p=.000; coef. = 0.152, p=.0001 respectively), whereas disappointment and blame decrease in Bs’ compliance (coef. = -0.113, p=.001; coef. = -0.071, p=.037 respectively).

**References**

1. Binmore, K., Morgan, P., Shaked, A., and Sutton, J. Do people exploit their bargaining power? An experimental study. Games and Economic Behavior. 1991; 3: 295–322.
2. Reuben, E. and F. van Winden. Social Ties and Coordination on Negative Reciprocity: The Role of Affect. Journal of Public Economics. 2008; 92: 34-53.

**Instructions (Baseline)**

*(translated from Italian)*

Good morning, thank you for participating in this experiment. You are taking part into a study on economic decisions. During the experiment, you can, depending on your decisions and on other participants’ decisions, earn an amount of money in addition to the 2 euros you will receive for showing-up on time. The answers you give and the choices you make will be totally anonymous. The experimenters will not be able to associate your choices and your answers to your name.

During the experiment you cannot communicate with other participants (otherwise you would be excluded from the experiment) and you should be very careful in reading the instruction that will appear on your screen and will be read out by one of the experimenters. If you have any questions, please ask the experimenters.

Your earnings will be calculated in tokens; each token will be converted in euros at the following ratio: 1 token = 0,01 euros.

At the end of the experiment, you will be asked to fill a short questionnaire; afterwards, we will proceed with the payment, that will occur in cash.

THE PARTICIPANTS

In this experiment there are in total 24 participants, which are divided into 8 groups with 3 members each. In each group, one subject will have the “A” role and two subjects will have the “B” role: the assignment of the role will be made by the computer at random. The role assigned and the group composition will be the same for the whole experiment. Therefore you will always interact with the same two people, but you do not know their identity, and they do not know your own identity.

The experiment is composed by 10 rounds. Each round is made of 4 Stages. During Stage 1, both subjects A and B will answer to three general-knowledge multiple-choice questions (with four possible answers each, being correct only one of them): if you pick the correct answer, you will earn 40 tokens. Furthermore, you have 25 seconds to answer each question: you will earn 1 token for each second you save.

In Stage 2, subjects A receive a feedback on how many tokens each subject B in their group did earn and can decide to how many tokens they want to withdraw from each subject B in their group. The request must be limited to the tokens earned by B in that round.

In Stage 3, subjects B answer to the (possible) request by A choosing to give A a certain amount of tokens (between zero and the number of tokens A has requested).

In Stage 4, subjects A receive a feedback on the number of tokens each B in their group decided to give them and can choose to reduce the number of questions subjects B can answer in the next round or leaving it unchanged. The cost of eliminating each question is 10 tokens.

# **Examples of questions from the multiple choice, general knowledge questions**

*(translated from Italian)*

1) What is the anemometer?

1. It measures the intensity of precipitations
2. It measures the speediness of the wind
3. It measures the heath dispersed in the ground
4. It measures the pureness of a diamond

2) The birth rate is the ratio between:

1. The number of births multiplied by 1,000 and the total population in a specific moment;
2. The number of births multiplied by 100 and the total population in a specific moment
3. The number of births multiplied by 10,000 and the total population in a specific moment
4. The number of births multiplied by 100,000 and the total population in a specific moment

3) If we observe a “Triforio”, it means we are:

A. In a square in Siena

1. On the edge of a fountain
2. In a church
3. On a tower

# **Post-experimental questionnaire**

*(translated from Italian)*

1) Gender

2) Age

3) Major

4) Now we ask you to state the level of intensity you experienced the following emotions during the experiment. The scale is 1 to 7, where 1 indicates “zero intensity” and 7 indicates “maximum intensity”:

 Happiness: _____

 Guilt: ______

 Shame: _______

 Surprise: _________

 Envy: _______

 Anger: _________

 Sadness: ________

 Irritation: ________

 Pride: _______

 Gratitude: ______

 Admiration: _______

 Fear: ______

 Blame: _______

 Disappointment: ________

 Grief: ________

 Indignation: __________

5) Now we ask you to state which level of request (as percentage of the tokens earned by B) from A to B you evaluate as fair: _______
